# PediDraw: A web-based tool for drawing a pedigree in genetic counseling

**DOI:** 10.1186/1471-2350-8-31

**Published:** 2007-06-08

**Authors:** Min He, Wei Li

**Affiliations:** 1 Key Laboratory of Molecular and Developmental Biology, Institute of Genetics and Developmental Biology, Chinese Academy of Sciences, Datun Road, Chaoyang District, Beijing 100101, China; 2 Graduate School of Chinese Academy of Sciences, 19 Yuquan Road, Beijing 100039, China

## Abstract

**Background:**

Drawing a pedigree is a prerequisite in genetic counseling. In medical records, a pedigree is useful to document the family history of the patient. Drawing a pedigree is also necessary in collecting genetic resources for medical research such as positional cloning. Currently, most pedigrees are drawn by hand or by drawing software. Due to the special requirements in a standardized pedigree, generating a pedigree by these methods is usually time-consuming and requires professionals. This limits the usage of a pedigree as demanded in remote diagnosis or online counseling from the counselees to send an electronic pedigree.

**Results:**

We developed an online pedigree drawing tool, PediDraw, which enables users to generate pedigrees after inputting the family information step-by-step on web. It outputs a pedigree or table to present a family history to the counselors.

**Conclusion:**

PediDraw is a user-friendly web-based drawing tool. It is accessible via Internet.

## Background

A pedigree is a genealogical graph or table which records the relationship of families by degrees. It is essential to genetic counseling. Drawing a pedigree is regarded as a trained skill of genetic counselors to obey the general rules of pedigree nomenclature [1]. Genetic counselors usually draw the pedigrees by handwriting or with the assistance of some commonly used drawing software such as Microsoft PowerPoint. This handwriting procedure is usually time-consuming and may lead to a mess when modifying. It will take a lot of time to align the icons and to annotate the pedigree when using drawing software such as Microsoft PowerPoint. In medical records, the family history is required to document and trace the inheritability of certain kind of disease. As the usage of electronic medical files is more popular in medical practice, the inconvenience in drawing a pedigree by hand or by current drawing software precludes the general practitioners from using the electronic files. In medical research, a detailed pedigree is required in genetic mapping or linkage analysis, which calls for a simple tool to draw pedigrees [2]. More recently, online genetic education and genetic counseling websites have been developed in the Internet era [3]. This service requires a simple drawing tool for the online genetic counselees without professional training.

To meet the above needs, several tools for drawing a pedigree or record the family history have been developed. Most of them are not web-based and can not be freely online accessible by users (such as the commercial pedigree drawing programs Cyrillic, Progeny) as listed in the category of "Pedigree Drawing Programs/Software Reviews" compiled by a genetic counselor, Debra Collins, at University of Kansas Medical Center, although it is not comprehensive [4]. A well-designed web tool to document a family history, My Family Health Portrait, is developed by the US Department of Health and Human Services [5]. As a tool from the US Surgeon General, it is easy to learn by Internet users, but does not output a standardized pedigree used by genetic counselors. It is not user-friendly when inputting multiple family members. Markinen et al. [6] have developed a high-throughput pedigree drawing tool, which is suitable for large families, but not for a standardized pedigree as recognized by genetic counselors [1]. Some pedigree drawing or editing program is limited by operating system, such as Madeline [7] is supported by Linux, Pedigree-Draw [8] is limited to Macintosh users, while Pelican [9] is a Java-based software tool. Here we designed a pedigree drawing tool that is online accessible and user-friendly, which can be easily implemented in online genetic counseling or remote medical practice by obeying the general rules of a standardized pedigree nomenclature.

## Implementation

### Programming

#### Programming languages

HyperText Markup Language (HTML), JavaScript (JS), Cascading Style Sheets (CSS), and Personal Home Page (PHP). All the data pages for storing the input information in a pedigree were converted to Structures Query Language (SQL) files based on the MySQL database system.

#### Programming editors

EditPlus text editor, Macromedia Dreamweaver MX, Photoshop CS.

### Copyright and server

PediDraw™ (version 1.0) is copyrighted by The National Copyright Administration of People's Republic of China. It is hosted in HP ProLiant ML150 dual Xeon 2.80-GHz server (Hewlett-Packard, Shanghai, China). It is running in Microsoft Windows XP environment. Apache provides a secure, efficient and extensible server for HTTP services.

## Results

The following five modules are the operations the users have to complete sequentially.

### Registration and login

Before starting a pedigree drawing, a user has to register. When the registration is finished, the user can login by using his/her username and password. When the user re-logins, the profile that was input the last time is displayed.

### User's information

The first sheet inputs the registered user's information including age, sex, affection status, marital status. If the user is married, (s)he may specify the affection and survival status of her/his spouse and children. The user may edit the existing information when (s)he re-logins. When the user is not sure of the information, (s)he could check any box first, and modify or delete it later on. When finished, click the "Next" button for the next step.

### Ancestors' information

This sheet records the affection status and survival status of any user's ancestors, including parents and grandparents. When finished, click the "Next" Button for proceeding.

### Family member's information

In this sheet, PediDraw allows the user to input all the information (name, affection status, survival status) of her/his family members, including the user's brothers/sisters, and the brothers/sisters of the user's father and mother. To avoid the confusion, the name of a family member could be real or nickname, but any two members in the family should have different names. To extend the family members, underneath each selected family member, it allows the user to input the affection status and survival status of each family member's spouse and children. The information can be modified or deleted when editing. Choose "Modify" or "Delete" when the information of a member is filled. When verified, click the "Add" button, the added member will be showing below the main table in orange color. When all the family members are specified, click "CONFIRMATION" button above the main table to finish this step.

### Submit and draw

**Figure 1:**
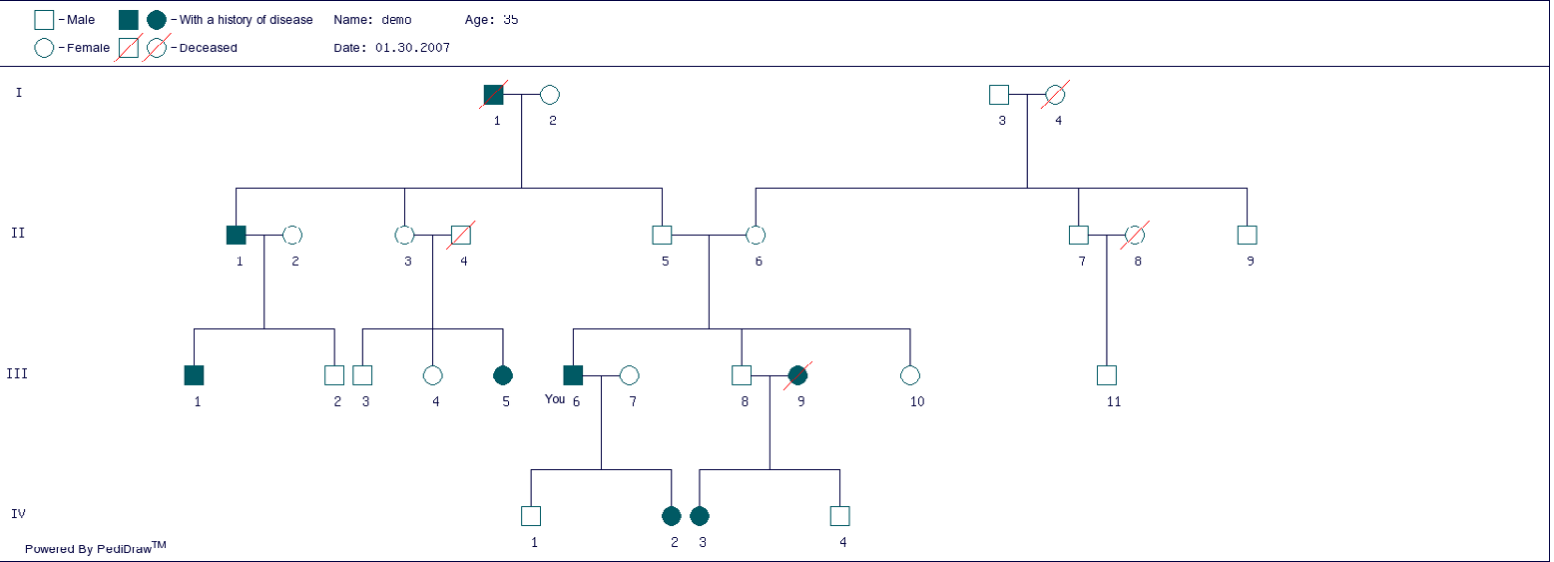
**A hypothetical clinical pedigree tree with migrane**. When choosing "Output type 2", the Table 1 is converted to a four-degree graph connected by the relationship lines. The symbols used in this graph are accepted in a standardized pedigree as described by Bennett, et al. [1]

Here displays a confirmation table of all the input information of the user's pedigree. In the table each member of the pedigree has been assigned an ID number automatically (Table 1). Underneath this table, supplement information can be added in the "REMARKS" block, such as the name of the disease, the symptoms of the disease, designation of a proband, consanguineous marriage or twins by using the ID numbers (Table 2). When finished, click the "Submit & Draw" button to go to the final step. Output type 1 is a pedigree table together with the remark table (Tables 1 and 2). When clicking the table icon, the output table could be saved or printed as a Microsoft Word file ("*.doc"). Output type 2 is a pedigree tree which could be saved as a "*.bmp" file or a "*.gif" file or printed directly when clicking the tree icon (Fig. 1). The combination of a table and a tree provides detailed information of a family history. This makes the appearance of a pedigree cleaner than the sample pedigree as shown in the website of National Society of Genetic Counselors [10]. To protect the privacy of a user's family information, the user can eliminate the submitted data from our database after saving a table or graph.

**Table 1:** A hypothetical clinical pedigree table with migrane.

Name: demo	Age: 35	Date: 01.30.2007	Powered by	
PediDraw™				

id	member	name	affected	alive

1	father's father		yes	no
2	father's mother		no	yes
3	mother's father		no	yes
4	mother's mother		no	no
5	father		no	yes
6	mother		no	yes
7	you	demo	yes	yes
8		Spouse	No	Yes
9		Son1	No	Yes
10		Daughter1	Yes	Yes
11	Father's Younger Brother	David	Yes	Yes
12		Spouse	No	Yes
13		Son1	Yes	Yes
14		Son2	No	Yes
15	Father's Elder Sister	Kate	No	Yes
16		Spouse	No	No
17		Son1	No	Yes
18		Daughter1	No	Yes
19		Daughter2	Yes	Yes
20	Mother's Elder Brother	Peter	No	Yes
21		Spouse	No	No
22		Son1	No	Yes
23	Mother's Younger Brother	Robert	No	Yes
24	Elder Brother	Alex	No	Yes
25		Spouse	Yes	No
26		Son1	No	Yes
27		Daughter1	Yes	Yes
28	Younger Sister	Jane	No	Yes

**Table 2:** Additional information to the pedigree in Table 1.

**Remarks**	
Type of disease:	migrane
Clinical symptoms:	starting at teenage, about once a week in frequency, pulsating headache, without aura, weather related, with hyperlipidemia.
Proband:	7
Consanguineous marriage:	15 and 16; 24 and 25
Twins:	no

PediDraw is featured with "Help" information in each step. The major operations of a user are to make selections from the icons. A user may return to the previous page by using the backward button in the tool bar. All these features enable an untrained counselee to learn how to use PediDraw easily.

## Discussion

PediDraw provides an online drawing tool for a pedigree that is recognized by any genetic counselors as it obeys the standardized nomenclature of a pedigree [1]. Although the current version (1.0) only allows the user to draw a four-degree pedigree, the number of family members is fairly unlimited, as the number of brothers/sisters is unlimited. Each core family in the pedigree is limited to eight members: a couple, three sons, and three daughters. This may limit the input of family members from large families. To solve this problem, due to the unlimited number of brothers/sisters, the user may assume one person with many brothers/sisters as the registered user or split a large family into small families when necessary.

As described in the "Results" section, PediDraw allows the user to choose either a pedigree table or a pedigree tree for output, which can be saved in a computer or printed directly. This feature makes it feasible to send an electronic file to a genetic counselor. Currently, the Chinese version of PediDraw is implemented in the online genetic counseling in China Genetic Counseling Network (CGCN) [3]. In CGCN, the counselors can review the pedigrees sent together with their questions. The other application of PediDraw is to document the families recruited in a program of collecting Chinese genetic resources. Some immortalized cell lines from this collection were deposited at the Foundation Jean Dausset (CEPH) for the Human Genome Diversity Project [11].

## Conclusion

PediDraw is providing a user-friendly web-based pedigree drawing tool. It has two optional output formats, a table and a graph. It is readable by any genetic counselors as PediDraw applies the standardized rules of drawing a pedigree in genetic counseling.

## Availability and requirements

PediDraw provides an open access [12] to any Internet user after registration following the steps as described in the "Registration and login" section. The PediDraw™ is registered in The National Copyright Administration of People's Republic of China (No. 2006SRBJ2033). All rights are reserved. PediDraw is currently run on Intel based PCs. It is compatible with most browsers and operating systems.

## Competing interests

The author(s) declare that they have no competing interests.

## Authors' contributions

MH and WL designed the study. MH and WL wrote and tested the software. WL wrote the paper. All authors read and approved the final manuscript.

## Pre-publication history

The pre-publication history for this paper can be accessed here:


